# Functional Regeneration of the Sensory Root via Axonal Invasion

**DOI:** 10.1016/j.celrep.2019.12.008

**Published:** 2020-01-07

**Authors:** Ev L. Nichols, Cody J. Smith

**Affiliations:** 1 Department of Biological Sciences, University of Notre Dame, Notre Dame, IN, USA; 2 Center for Stem Cells and Regenerative Medicine, University of Notre Dame, Notre Dame, IN, USA; 3 Lead Contact

## Abstract

Regeneration following spinal root avulsion is broadly unsuccessful despite the regenerative capacity of other PNS-located nerves. By combining focal laser lesioning to model root avulsion in zebrafish, time-lapse imaging, and transgenesis, we identify that regenerating DRG neurons fail to recapitulate developmental paradigms of actin-based invasion after injury. We demonstrate that inducing actin reorganization into invasive components via pharmacological and genetic approaches in the regenerating axon can rescue sensory axon spinal cord entry. Cell-autonomous induction of invasion components using constitutively active Src induces DRG axon regeneration, suggesting an intrinsic mechanism can be activated to drive regeneration. Furthermore, analyses of neuronal activity and animal behavior show restoration of sensory circuit activity and behavior upon stimulating axons to re-enter the spinal cord via invasion. Altogether, our data identify induction of invasive components as sufficient for functional sensory root regeneration after injury.

## In Brief

Dorsal root ganglion (DRG) sensory axons are unable to regenerate into the spinal cord after injury. Nichols and Smith demonstrate in zebrafish that injured DRG axons do not initiate actin-based invasion components during re-entry into the spinal cord. Pharmacological and cell-autonomous genetic manipulations that promote actin-mediated cell invasion to restore sensory behavior.

## Graphical Abstract

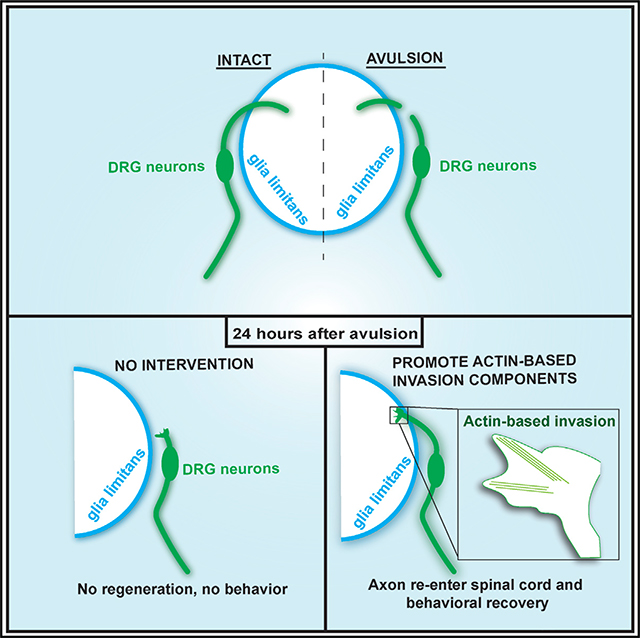

## INTRODUCTION

The peripheral nervous system (PNS) can regenerate following injury (Ertürk et al., 2007; Gribble et al., 2018; Rosenberg et al., 2012, 2014). One exception is the dorsal root following avulsion injuries in which the peripheral nerve root is torn from the CNS (Hoeber et al., 2017; Di Maio et al., 2011; Ramón-Cueto and Nieto-Sampedro, 1994). In humans, these injuries occur in adulthood following severe trauma or in neonates at birth. The latter type, obstetrical brachial plexus injury (OBPI), occurs in 1 in 3,000 live human births, leaving patients with permanent sensorimotor defects (Thatte and Mehta, 2011). Across phylogeny, root avulsions do not fully recover because PNS-located sensory axons in the dorsal root ganglion (DRG) cannot re-enter the spinal cord. Attempts at aiding DRG axon re-entry into the CNS have been successful in the laboratory: implantation of stem cells or glia, addition of ectopic growth factors to the dorsal root, inhibition of the glial scar, and peripheral nerve injury (Hellal et al., 2011; Hoeber et al., 2017; Neumann and Woolf, 1999). However, each of these approaches faces important drawbacks for clinical use.

Here, we explore the relationship between regenerating DRG axons following OBPI-like injuries and developmental paradigms that drive pioneer axon dorsal root entry zone (DREZ) entry in larval zebrafish. We show that regenerating axons do not form invasive actin concentrates to re-enter the spinal cord. However, stabilization of invasion components with both pharmacological and cell-autonomous interventions promotes DRG axon spinal entry after avulsion. Promotion of sensory regeneration via cell invasion also rescues animal function at the circuit and behavioral levels. Altogether, our data identify cell invasion as a mechanism of regeneration following neural injury.

## RESULTS

### Sensory Root Regeneration Fails Because Axons Are Unable to Invade the Spinal Cord

**Figure 1.**
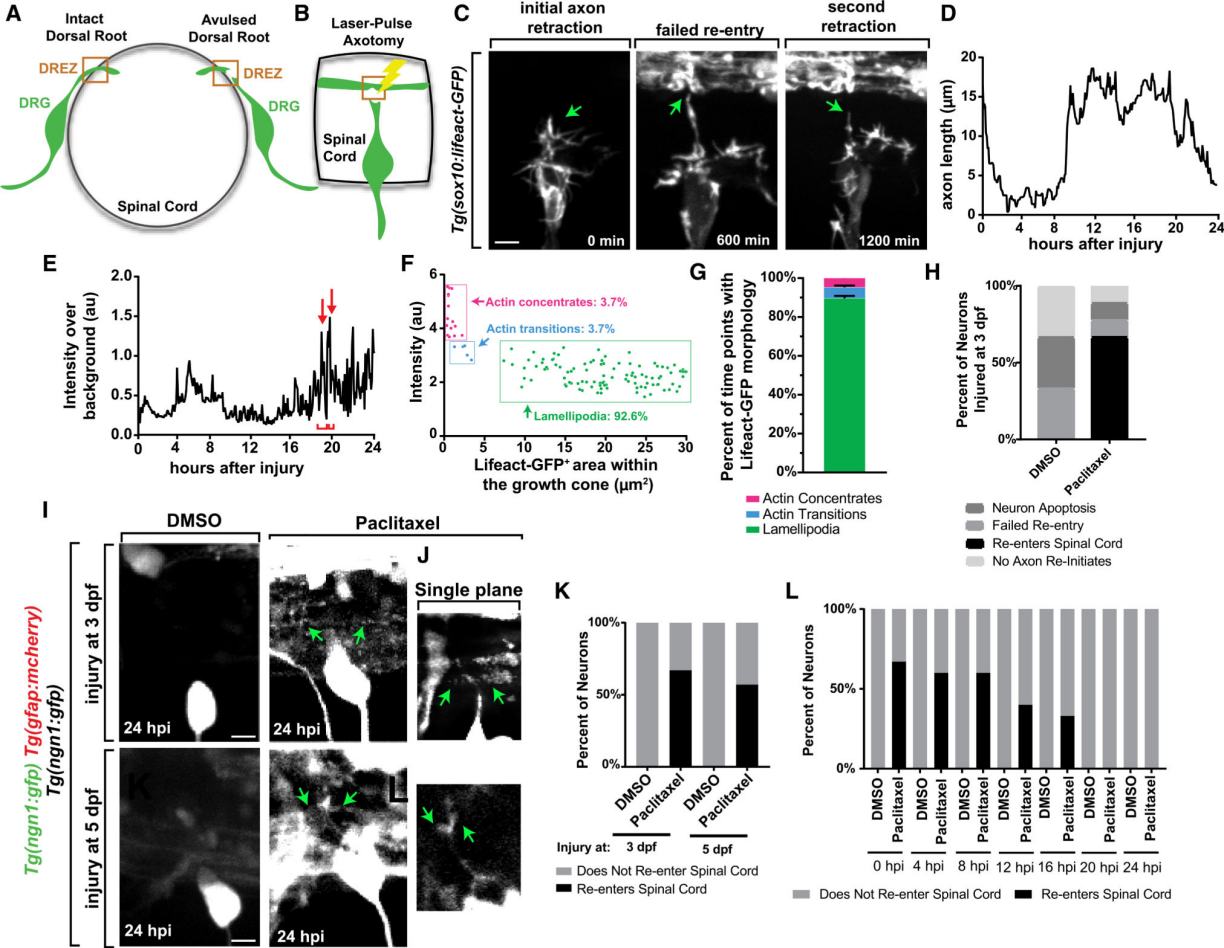
Taxol Rescues DRG Axon Spinal Entry after Avulsion-like Injury. (A) Cross-section diagram of an intact and avulsed dorsal root. (B) Diagram of experimental model. At 3 dpf, a dorsal root is axotomized and time lapse imaged. (C) Z-projection time-lapse images of an avulsed DRG in a *Tg(sox10:gal4); Tg(uas:lifeact-gfp)* animal. Green arrows denote the growth cone. (D) Representative graph of axon length following injury. (E) Representative quantification of Lifeact-GFP intensity at the growth cone throughout regeneration. Red arrows represent actin structures, and brackets represent the duration of Lifeact peaks. (F) Representative scatterplot of the growth cone area and average Lifeact-GFP intensity during regeneration. Each time point is represented by a point. (G) Percentage of time points for which the regenerating growth cone displayed each actin organization. n = 5 DRG. (H) Outcomes of regeneration in 3 dpf injuries. n = 6 DRG per treatment. (I and J) Z-projection (I) and single-plane (J) images of *Tg(ngn1:gfp); Tg(gfap:nsfb-mcherry)* animals treated with DMSO or Taxol at 24 hpi. Injures at 3 and 5 dpf. Green arrows denote regenerated axons. (K) Outcomes of DRG injured at 3 or 5 dpf. n = 6 at 3 dpf, 7 at 5 dpf. (L) Outcomes of avulsed DRG treated with DMSO or Taxol at varying times after injury. n = 5 per treatment for 0–8 hpi, 6 per treatment for 12–24 hpi. Scale bar, 10 μm.

The sensory root does not regenerate following avulsion injuries (Figure 1A; Hoeber et al., 2017; Di Maio et al., 2011; Ramón-Cueto and Nieto-Sampedro, 1994). However, attempted regeneration by DRG axons has not been imaged in totality, limiting our understanding of mechanisms underlying failed regeneration. To provide mechanistic insight into this process, we used a recently developed zebrafish model for avulsion-like injuries (Green et al., 2019). We used focal laser-pulse lesioning (Ablate) to axotomize single DRG axons in the PNS at 3 days post-fertilization (dpf) (Green et al., 2019; Figure 1B). This laser specifically targets select diffraction-limited regions with scalable laser pulse energies to minimize damage to surrounding tissue (Green et al., 2019). A sensory root injury at this zebrafish age corresponds with OBPI cases in human development, namely, the onset of myelination and the expansion of nerve roots (Green et al., 2019).

To visualize actin in the DRG, we expressed Lifeact-GFP in DRG cells using *sox10* promoter elements with *Tg(sox10:gal4); Tg(uas:lifeact-gfp)* animals (Helker et al., 2013; Hines et al., 2015). After performing a single axotomy in each animal, we imaged actin dynamics in regenerating neurons every 5 min for 24 h. Within 1 h after injury, the lesioned axon retracts to the DRG soma (Figure 1C; Video S1). This retraction is reminiscent of injured spinal axons (Bradke et al., 2012; Kerschensteiner et al., 2005; Windle, 1980). The DRG axon then re-extends dorsally toward the DREZ. After contacting the DREZ, the regenerated axon again retracts back to the soma (Figures 1C and 1D; Video S1). One day after injury, the soma lacks a dorsal axon while maintaining a peripheral axon. These axon-less cell somas do not undergo apoptosis through 4 days post-injury.

In development, DRG pioneer axons enter the spinal cord by concentrating growth cone actin to form invadopodia (Nichols and Smith, 2019). These invadopodia are required to cross the spinal cord boundary, or glia limitans. A glial scar forms at the glia limitans following avulsion, so we hypothesized that DRG regeneration fails because the regenerating axon does not form invadopodia (Hellal et al., 2011; Hoeber et al., 2017; Mokalled et al., 2016). To test this, we traced Lifeact-GFP intensity in the re-extending growth cone in our videos and observed short but sharp spikes in Lifeact-GFP intensity at the DREZ (Figure 1E). Each regenerating axon initiated multiple (3.2 ± 0.2) actin peaks, but these spikes were transient; that is, they were only present for 26.31 ± 2.68 min (n = 6 DRG). Compared with developmental invasion in which actin concentrates persist for ∼40 min, regenerating axons form short-lived actin invasion formations.

To understand the relationship between growth cone and F-actin, we quantified the area and average Lifeact-GFP intensity of the growth cone at each point in its navigation and generated scatterplots (Figures 1F and 1G). In these graphs, each point represents a single time point. A small portion of time points had a small growth cone but a large Lifeact-GFP signal, similar to invasive structures in development (Nichols and Smith, 2019). Comparison of these time points with Figure 1E revealed that these time points were the identified actin concentrates. We next observed a group of time points for which the growth cones were slightly smaller and displayed less Lifeact-GFP. These were the time points temporally adjacent to actin concentrates, which we termed actin transitions. The remaining time points displayed dispersed actin (a larger growth cone area and lower Lifeact-GFP). This analysis indicates that the growth cone dynamically adjusts its actin organization. These cytoskeletal states include the initiation of actin rearrangements consistent with invasion, but these structures fail to stabilize.

### Pharmacological Stabilization of Invasion Rescues Axon Entry after Injury

If DRG regeneration fails because axons do not form invasion components, then stabilization of invasion components should promote axon spinal re-entry. We tested this by treating avulsed animals with the microtubule stabilizer paclitaxel (Taxol), which stabilizes invasion components in DRG pioneer axons and proinvasive cells (Nichols and Smith, 2019; Quintavalle et al., 2011). We visualized sensory neurons by expressing GFP under the *ngn1* promoter, *Tg(ngn1:gfp)*, and the glia limitans by expressing mCherry with *gfap* promoter elements, *Tg(gfap:nsfb-mcherry)* (McGraw et al., 2008; Smith et al., 2016). We performed avulsion-like injuries at 3 dpf, immediately treated with Taxol, and time lapse imaged for 24 hours post-injury (hpi). 67% of injured Taxol-treated axons did not retract a second time and instead formed anterior and posterior projections (n = 6 DRG; Figures 1H–1K). To ask whether they re-entered the spinal cord, we digitally rotated these images 90°and detected a GFP^+^ axonal puncta inside of the mCherry^+^ glia limitans (Figure S1A). 0% of DMSO-treated control axons re-entered the spinal cord and instead exhibited no dorsal axon (n = 6 DRG; Figures 1H–1K). We considered the possibility that Taxol allows for spinal re-entry by preventing the initial retraction of the axon but ruled this out because our time-lapse videos still demonstrated Taxol-treated axons retracting to the DRG before regenerating dorsally (Figures S1B–S1E). It is also possible that Taxol-mediated regeneration is specific to a 3 dpf injury, not older ages. To test this, we repeated this experiment in 5 dpf animals. In this experiment, 57% of Taxol-treated axons regenerated into the spinal cord, comparable with regeneration in 3 dpf animals. This supports the conclusion that Taxol-mediated regeneration could extend to later ages (n = 7 Taxol, 7 DMSO; Figures 1H, 1I, 1K, and S1F).

Our data demonstrate that the axon will retract toward the DRG after a failed attempt at re-entry. This suggests that there may be a window for Taxol treatment to successfully promote regeneration. To test this, we created avulsion-like injuries at 3 dpf and applied treatment of Taxol at intervals of 4 h after injury. We then imaged these animals at 48 hpi to assay for regeneration. Similar levels of regeneration can be achieved with Taxol treatment starting from 0–8 hpi (n = 5 DRG per treatment per time; Figures 1L and S1G). Rates of successful regeneration begin to steadily decrease starting at 12 hpi treatments and falling to 33% success by 16 hpi (n = 6 DRG per treatment per time). All attempts at regeneration with Taxol treatment after 20 hpi were unsuccessful (n = 6 DRG per treatment per time). These measures correspond with the approximate time of axon retraction in Figure 1D, indicating that the second axonal retraction rapidly decreases the probability of re-entry. Altogether, these data suggest that treatment between 0 and 16 hpi is required for successful Taxol-induced regeneration.

### Taxol Stabilizes Invasion Components to Drive Spinal Cord Re-entry following Injury

**Figure 2.**
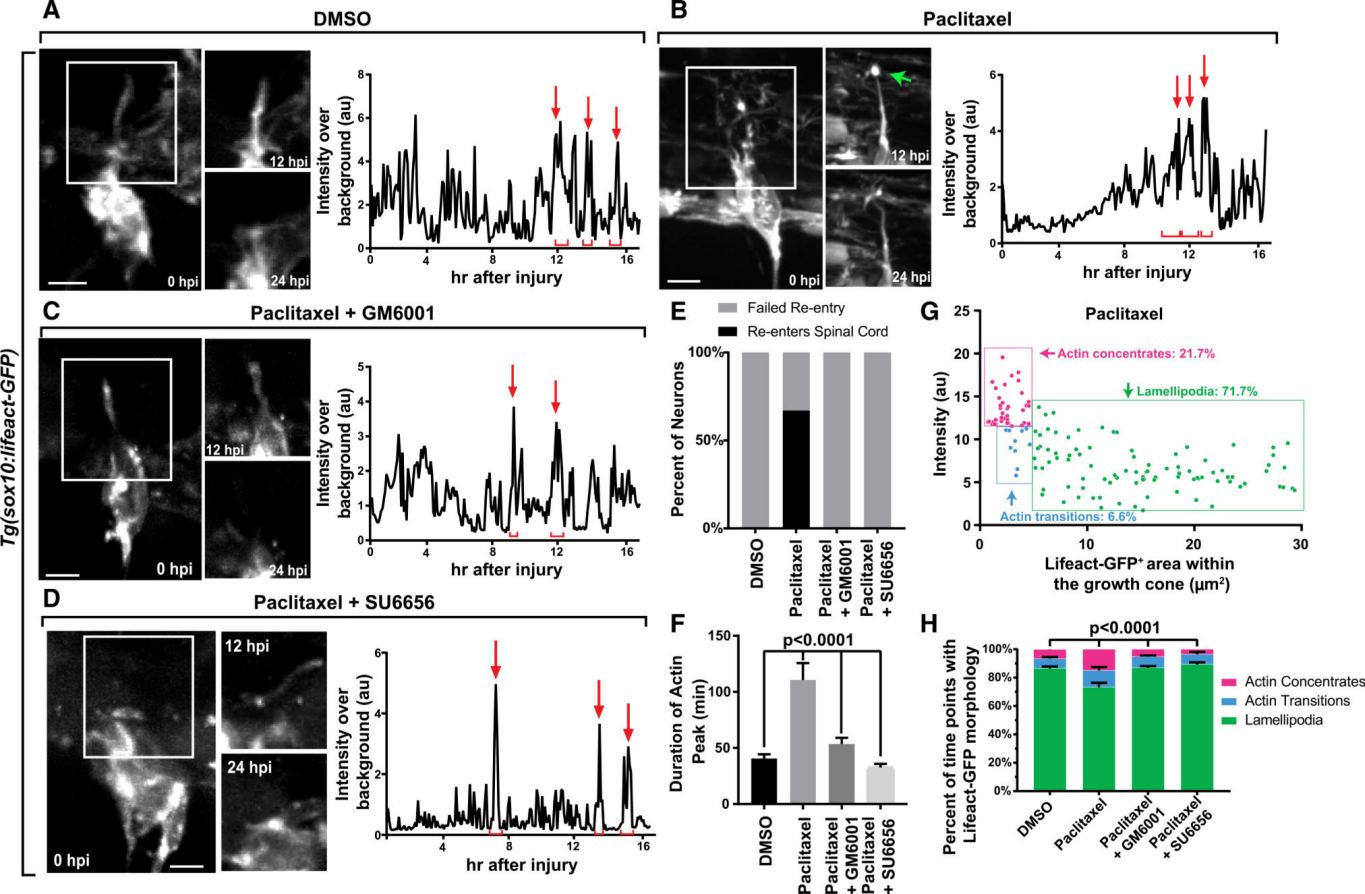
Taxol Induces Actin-Rich Invasion Components during Spinal Re-entry. (A–D) Z-projection time-lapse images of avulsed DRG in *Tg(sox10:gal4); Tg(uas:lifeact-gfp)* animals treated with DMSO (A), Taxol (B), Taxol+GM6001 (C), and Taxol+SU6656 (D). Graphs show Lifeact-GFP intensity tracings during regeneration. White boxes denote insets of the growing axon and growth cone regions. The green arrow denotes a stable invasive structure. Red arrows and brackets represent peaks and durations of peaks of Lifeact-GFP. (E) Graph of spinal re-entry in axons treated with DMSO, Taxol, Taxol+GM6001, and Taxol+SU6656. (F) Duration of actin concentrates in DMSO axons (n = 7), Taxol axons (n = 6), Taxol+GM6001 axons (n = 6), and Taxol+SU6656 axons (n = 4). (G) Representative scatterplot of growth cone area and average Lifeact-GFP intensity during regeneration in an axon treated with Taxol. Each time point is represented by a point. (H) Percentage of time points for which the regenerating growth cone displayed each actin organization. Tukey’s HSD was used in (D) and two-way ANOVA was used in (H). Scale bar, 10 mm.

To test potential mechanisms for Taxol-mediated regeneration, we visualized actin in regenerating axons using *Tg(sox10:lifeact-gfp)* animals and subjected them to our avulsion model. We considered that Taxol promotes regeneration by initiating invasion components but ruled this out, because Lifeact-GFP peaks initiate indistinguishably in DMSO and Taxol-treated animals (n = 6 DRG per treatment; Figures 2A and 2B; Video S2). When we compared the time after injury that these spikes initiated and the total number of spikes between treatments, we also did not detect differences (Figures S2A and S2B). We next considered that invasion of these regenerating neurons was dependent on stabilization of actin-based invasion components by measuring the duration of Lifeact-GFP peaks (Figure 2F). Actin-based invasion components in DMSO-treated animals formed for 40.59 ± 3.71 min (n = 17 concentrates) compared to Taxol-treated animals that stabilized for 110.7 ± 14.96 min (n = 20 concentrates), consistent with regeneration dependent on invasion stabilization. We also repeated our analysis of growth cone size and Lifeact-GFP intensity. Taxol-treated growth cones navigated with actin concentrates and actin transitions more than DMSO-treated growth cones (p < 0.0001; Figures 2G, 2H, S2C, and S2D). The simplest explanation for these data is that Taxol promotes invasion of regenerating sensory axons via stabilization of actin-based invasion.

Invasion relies on coordination of Src-driven actin invasive structures and matrix metalloproteinases (MMPs) (Clancy et al., 2015; Santiago-Medina et al., 2015; Sedgwick et al., 2015). If Taxol is driving regeneration via stabilization of invasive structures, then inhibition of these other components should abolish this regeneration. To test this, we cotreated avulsed animals with GM6001 and Taxol, as well as SU6656 and Taxol. GM6001 is a pan-inhibitor of MMPs, and SU6656 inhibits Src family kinases. After injury, these axons retracted, re-extended, and then retracted a second time (n = 6 for GM6001, 4 for SU6656; Figures 2C and 2D; Video S3). As such, none of the cotreated axons re-entered the spinal cord (Figure 2E). Like DMSO-treated axons, cotreated axons did not form robust actin concentrates but rather transient actin accumulations (n = 20 GM6001 concentrates, 14 SU6656 concentrates; p < 0.0001, one-way ANOVA; Figure 2F). We also compared the number of actin accumulations (p = 0.8341, one-way ANOVA) and the time of initiation (p = 0.0892, one-way ANOVA) but did not observe differences between treatments (Figures S2A and S2B). These observations were supported by measurements of the growth cone size and Lifeact-GFP intensity at individual time points (p < 0.0001, two-way ANOVA; Figures 2H, S2D, and S2E). Altogether, these data support the hypothesis that Taxol stabilizes invasive components in regenerating axons, whereas inhibition of molecules necessary for invasion counteracts Taxol’s pro-regenerative effects.

To dissect the mechanism of Taxol-mediated regeneration, we considered the possibility that Taxol could have dynamic effects on the growth cone independent of actin organizations. To do this, we measured the velocity of the growth cone during actin concentrates identified earlier. In DMSO axons, the axon navigated at −0.0758 ± 0.103 μm/min, indicating slight retreats from the DREZ. In Taxol-treated axons, the opposite occurred with, the axons extending at 0.142 ± 0.44 μm/min (p = 0.0037, Tukey’s honest significant difference [HSD]; Figure S2F). This suggests that Taxol confers a pro-growth state. However, the same effects on axon velocity were observed in cotreatment axons (GM6001, 0.0226 ± 0.037 μm/min; SU6656, 0.066 ± 0.051 μm/min) axons, despite lack of re-entry (p > 0.3213, Tukey’s HSD; Figure S2F). The simplest explanation for these data is that permissive growth cone extension by Taxol treatment is not sufficient for regeneration.

### Stabilization of Invasion Machinery Cell-Autonomously Promotes DRG Regeneration

**Figure 3.**
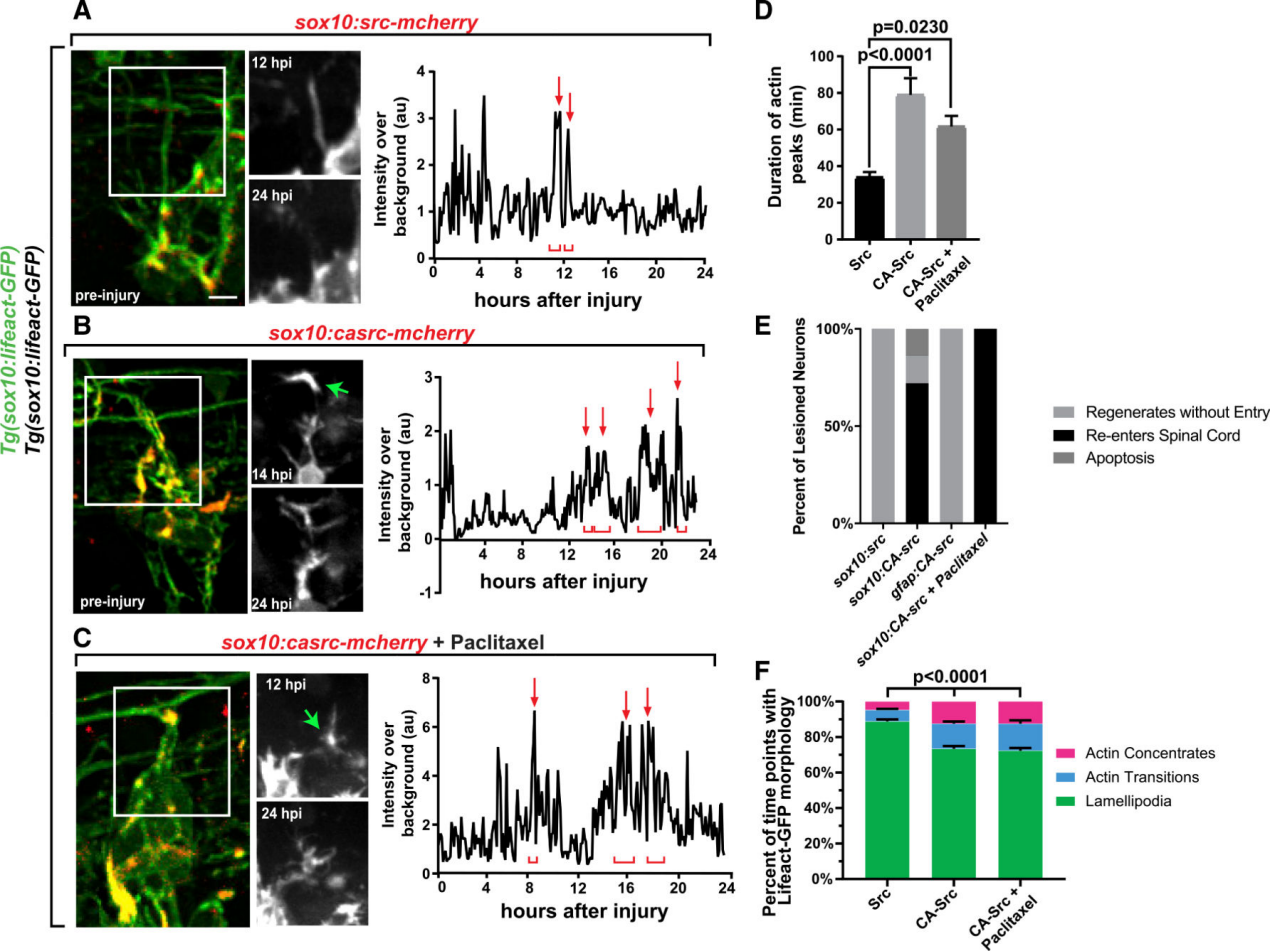
Induction of Invasion Components in DRG Neurons Promotes Regeneration. (A–C) Z-projection time-lapse images of avulsed DRG in *Tg(sox10:gal4); Tg(uas:lifeact-gfp)* animals expressing *uas:src-mcherry* (A), *uas:CA-src-mcherry* (B), or *uas:CA-src-mcherry* and treated with Taxol (C). Graphs show Lifeact-GFP intensity tracings throughout regeneration. The green arrow denotes a stable invasive structure. Red arrows and brackets represent peaks of Lifeact-GFP. (D) Actin concentrate duration in Src DRG (n = 6), CA-Src DRG (n = 6), and CA-Src+Taxol DRG (n = 5). (E) Regeneration outcomes in DRG expressing *sox10:Src, sox10:CA-Src, or sox10:CA-Src*+Taxol or radial glial expressing gfap:CA-Src. (F) Percentage of time points the regenerating growth cone navigated using each actin organization. Tukey’s HSD was used in (D) and two-way ANOVA was used in (F). Scale bar, 10 μm.

Previous studies have identified Taxol as a negative regulator of glial scar formation after dorsal hemisection (Hellal et al., 2011). This raises the possibility that induction of invasion components in regenerating axons could be secondary to glial scar inhibition. We sought to test this by inducing invasion cell-autonomously. Src activation has been shown to enhance cell invasion by stabilizing actin-rich invasion (Murphy and Courtneidge, 2011), so we hypothesized that cell-specific expression of constitutively active Src (CA-Src) in DRG cells would drive invasion in regenerating growth cones. Replacing an inhibitory tyrosine phosphorylation site with a phenylalanine (Y528F) in Src encodes CA-Src (Seiler et al., 2012). We expressed CA-Src fused to mCherry in DRG cells to drive expression under the *sox10* promoter. We also visualized Lifeact-GFP in DRG cells, created avulsion-like injuries, and imaged actin (n = 6 CA-Src, 6 Src; Figures 3A and 3B; Video S4). Src-mCherry overexpression was used as a control. CA-Src-expressing cells had actin peaks that stabilized for 78.95 ± 9.11 min (n = 19 concentrates). In controls, Lifeact-GFP peaks were present for 33.94 ± 2.86 min (n = 17 concentrates; Figure 3D). However, we observed no difference in number of peaks or time of peak initiation (Figures S3A and S3B). Likewise, CA-Src-expressing axons navigated more with actin concentrates and actin transitions compared with axons expressing Src (Figures 3F, S3D, and S3E). These data indicate that expression of CA-Src in regenerating axons is sufficient to restore actin-based invasion components in regenerating axons. To assay for re-entry, we stained 24 hpi animals for GFAP, a marker for the glial limitans. Using surface intensity plots to mark the edge of the glial limitans and the LifeactGFP^+^ axons, we detected that 71.4% of axons recrossed the glial limitans (Figures 3E and S3C). This is in contrast to Src-expressing axons, in which 0% re-entered the spinal cord (Figures 3A and 3E). To test the potential of CA-Src to promote regeneration in additional cell types, we expressed CA-Src under a *gfap* promoter and assayed for axonal regeneration. In these animals, 0% of axons regenerated into the spinal cord at 24 hpi (n = 7 animals; Figures 3E and S3F).

Our data thus far indicate two distinct mechanisms can promote actin-based invasion axonal regeneration: microtubule stabilization and Src activation. To test potential overlap in these pathways, we treated CA-Src axons with Taxol and assayed regeneration (Figure 3C). We detected no difference in actin dynamics compared with CA-Src expression alone (n = 5 DRG; Figures 3D, 3F, S3A, S3B, and S3G). However, axon entry measurements revealed that 100% of Taxol-treated CA-Src axons re-entered the spinal cord (n = 5 DRG; Figures 3E and S3H). These data suggest that Taxol and CA-Src redundantly promote invasive actin formations, a common determinant of DRG regeneration.

### DRG Axon Regeneration after Injury Restores Sensory Circuitry and Behavior

**Figure 4.**
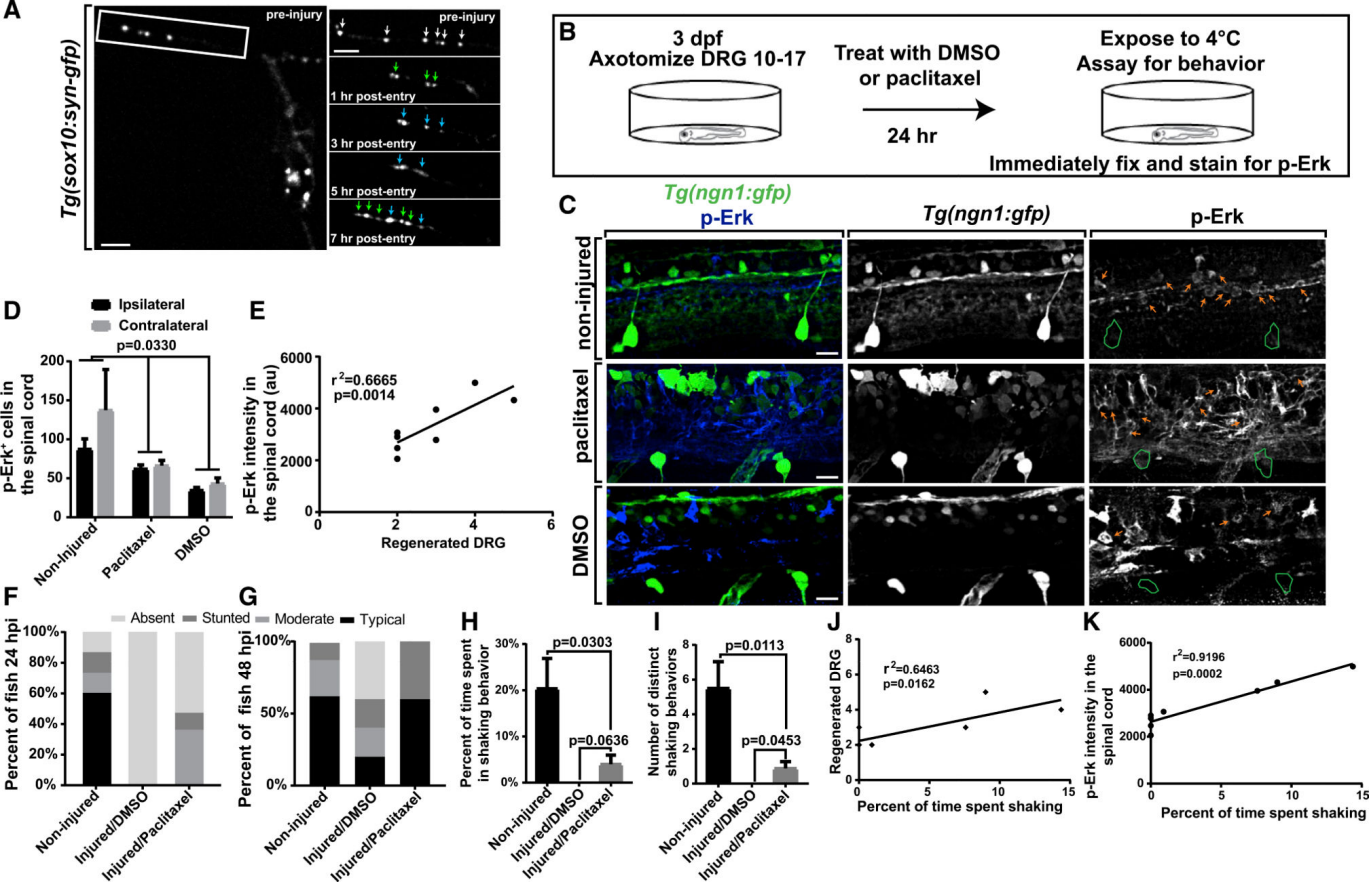
DRG Regeneration Restores Circuit and Behavioral Function. (A) Z-projection time-lapse images of a *Tg(sox10:syn-gfp)* animal treated with Taxol before and after injury. White box denotes the area of insets. White arrows denote Syn-GFP puncta before injury. Green arrows denote new Syn-GFP puncta after re-entry. Blue arrows denote stabilized Syn-GFP. (B) Diagram of circuit and behavioral analysis. (C) Z-projection images of *Tg(ngn1:gfp)* animals stained for pErk following exposure to 4°C without injury or 8 consecutive avulsions and DMSO or Taxol treatment. Green outlines denote the area of DRG. Orange arrows denote pErk^+^ spinal cells. (D) Number of pErk^+^ cells in the spinal cord on ipsilateral and contralateral sides of injury (n = 8 animals per treatment). (E) Taxol-treated animals show a positive correlation between regenerated DRG and pErk intensity in the spinal cord (n = 8 animals). (F and G) Behavior at 24 hpi (F) and 48 hpi (G) in animals without injuries, avulsions with DMSO, and avulsions with Taxol (n = 8 at 24 hpi, 5 at 48 hpi). (H and I) Percentage of time shaking (H) and number of shaking behaviors (I) in animals without injuries, avulsions, and DMSO treatment and without avulsions and Taxol treatment 24 hpi (n = 8 animals per treatment). (J and K) Taxol-treated animals show a positive correlation between percentage of time shaking and regenerated DRG (J) and between percentage of time shaking and pErk spinal intensity (K) (n = 8 animals). Two-way ANOVA was used in (D); linear regression was used in (E), (J), and (K); and Tukey’s HSD was used in (H) and (I). Scale bar, 10 μm.

To first determine whether invasion-mediated regeneration restores sensory function, we revisited our videos of regeneration with Taxol. All regenerated neurons formed anterior and posterior projections that traveled to their proper dorsal location by 24 hpi (Figure S4A). Furthermore, we tested whether spinal synaptic connections are restored following regeneration. To do this, we expressed GFP-tagged Synaptophysin (Syn-GFP) in DRG neurons in *Tg(sox10:syn-gfp)* animals, created an avulsion-like injury, treated with Taxol, and time lapse imaged. Soon after re-entry, Syn-GFP puncta were deposited along the regenerating axon. By 7 h post-entry, the synaptic puncta topographically resembled synapses preinjury (Figure 4A). These data indicate that regenerating sensory axons could still rapidly synapse with spinal cord neurons.

To test the function of the sensory circuit, we assayed for regeneration, activation of DRG and spinal neurons, and sensory-mediated behavior in the same animals (Figure 4B). To visualize regeneration, we used *Tg(ngn1:gfp)* animals and created eight consecutive avulsion-like injuries at 3 dpf. Avulsed animals were treated with Taxol or DMSO to dissect the effects of failed regeneration on behavior and neuronal activity. Non-avulsed animals were also used as controls. As a behavioral assay, we exposed animals to 4° C, which causes firing of DRG and spinal neurons during a hypothermic shivering behavior (Fosque et al., 2015). We gauged neuronal activation using MAP mapping at 24 hpi to visualize phosphorylated Erk (pErk) (Randlett et al., 2015).

We first measured regeneration and pErk levels in each of treatment group both ipsilateral and contralateral to the injuries. Measurements of pErk intensity of the spinal cord were elevated in unlesioned animals, consistent with circuit activity. Intensity of pErk in Taxol-treated animals was also elevated compared with DMSO-treated animals but less than unlesioned animals, consistent with a partially functioning sensory circuit (n = 8 animals per treatment; Figures 4C and 4D). We next compared the number of regenerated axons to the pErk intensity in spinal circuitry. Our data identify a strong positive correlation between the number of regenerated DRG and the number of spinal pErk (r^2^ = 0.6665, p = 0.0014, n = 8 animals; Figure 4E).

We considered the possibility that differences in pErk intensities could result from the injuries. To test this, we repeated the experiment and assayed for pErk levels in each treatment with exposure to 23° C. We did not detect differences in spinal pErk between treatments following exposure to 23° C (n = 5 animals per treatment; Figures S4B and S4C). Collectively, these data are consistent with the conclusion that regeneration of the sensory root partially restores circuit function.

We next measured behavioral responses to 4° C in the same animals to gauge sensory-mediated behavior. To quantify behavioral response, we measured the number of hypothermic shivers and the percentage of time spent shivering. These measurements were then used to classify behavioral responses into four categories: typical (3+ shivers and greater than12% shivering), stunted (1–3 shivers but 7%–12%), moderate (1–3 shivers and 1%–7%), or absent (no shivering). All DMSO-treated animals displayed absent behavioral response, whereas 60% of unlesioned animals displayed typical behavior. Taxol-treated animals displayed 37.5% moderate responses. A smaller portion exhibited stunted behavior (n = 8 animals per treatment; Figures 4F and S4D). Partial recovery is consistent when comparing the number of shivers and the percentage of time spent shivering individually (Figures 4H and 4I). Taxol-treated animals displayed partial recovery, but not as robust as unlesioned animals. We observed a positive correlation between percentage of time shaking and the number of regenerated axons in Taxol-treated animals (r^2^ = 0.6463, p = 0.0162; Figure 4J). Similarly, pErk intensity in spinal circuitry was strongly correlated with shivering (r^2^ = 0.9196, p = 0.0002; Figure 4K). Collectively, these data support the hypothesis that regeneration partially rescues sensory circuits and behavior by 24 hpi.

We next considered the possibility that recovery of sensory circuitry could continue past 24 hpi. To test this, we repeated the preceding experiment and assayed for behavior at 48 hpi. These measurements revealed similar phenotypes between unlesioned and Taxol-treated animals, indicating enhanced recovery at 48 hpi (n = 5 animals per treatment; Figures 4G and S4D). In contrast, behavioral recovery in DMSO-treated animals was unilateral and exaggerated to the uninjured side of the animal (Figure S4D). Nonetheless, the level of recovery of DMSO-treated animals remained less than that of Taxol-treated animals and non-injured animals. When we quantified behavioral dynamics, we did not detect differences in the number of shivers (p = 0.8031, one-way ANOVA) or the percentage of time shaking (p = 0.0783, one-way ANOVA) between unlesioned and Taxol-treated animals (Figures S4E and S4F). These data support the conclusion that invasion machinery can regenerate DRG axons and restore sensory circuitry and behavior.

## DISCUSSION

Many have proposed that the homeostatic state of mature neurons results in a fundamentally different intracellular milieu from developing neurons preventing regeneration (He and Jin, 2016; Di Maio et al., 2011; Perry et al., 2018). However, this explanation is likely insufficient. Our data suggest that invasive machinery is present in re-extending DRG axons, but these components cannot coordinate without pharmacological or molecular aid to allow the axon to extend past inhibitory cues at the injury site.

Although our data indicate that avulsion injures are an exception, zebrafish display enhanced regenerative capabilities, including spinal cord regeneration. In larval zebrafish, after complete spinal transection, functional recovery can be detected by 3 dpi, with complete recovery by 9 dpi (Briona and Dorsky, 2014). Alternatively, our data demonstrate behavioral recovery within 1–2 dpi in larval zebrafish after 8 consecutive avulsion-like injuries, a short timescale compared with other types of zebrafish regeneration.

Taxol has previously been identified as an enhancer of neuronal regeneration (Ertürk et al., 2007; Hellal et al., 2011; Hsu et al., 2017; Sengottuvel et al., 2011). Previous studies have identified it as a negative regulator of glial scar extracellular matrix (ECM) deposition following dorsal hemisection (Hellal et al., 2011). The corresponding decrease in ECM also correlated with axon regeneration, but these studies did not interrogate potential effects of Taxol on axonal invasion machinery. In the study above, cell-autonomous activation of invasion through expression of CA-Src is sufficient for sensory axon regeneration.

Mechanistically, these data also present a paradox: Taxol, a microtubule stabilizer, produces stabilized actin invasion. A molecular cascade connecting stable microtubules and actin nucleation could be responsible, because previous studies have shown a correlation between microtubule stability and Arp2/3 activity via HDAC6 and cortactin, a regulator of invadopodia (Shi et al., 2019). Alternatively, the microtubule plus-end protein EB1 could form a complex with actin regulators Src and Cortactin (Biosse Duplan et al., 2014). Altogether, the data presented here suggest an additional avenue for axon regeneration across restrictive boundaries by promoting cell invasion machinery to overcome physical limits to axonal regrowth.

## STAR * METHODS

### LEAD CONTACT AND MATERIALS AVAILABILITY

Further information and requests for resources and reagents should be directed to and will be fulfilled by the Lead Contact, Cody J. Smith (csmith67@nd.edu). All materials generated in this study are available upon request.

### EXPERIMENTAL MODEL AND SUBJECT DETAILS

#### Zebrafish

The University of Notre Dame Institutional Animal Care and Use Committee approved all animal studies. Zebrafish used in this study were: *Tg(sox10:gal4) (Hines et al., 2015); Tg(uas:lifeact-gfp)* (Helker et al., 2013), *Tg(ngn1:gfp)* (McGraw et al., 2008), and *Tg(gfap:nsfb-mcherry)* (Smith et al., 2016). Stable, transgenic lines were used in all experiments. Embryos were produced by pairwise matings and raised in darkness in egg water at 28° C. Zebrafish from 2–5 dpf were used for experiments. Healthy immune status zebrafish were utilized for experiments. Experiments utilized zebrafish embryos before sex is determined. Animals were subjected to one avulsion per animal with the exception of the behavioral experiments in Figure 4. All embryos were drug/test naive before the desired experiment.

### METHOD DETAILS

#### In Vivo Imaging

3-amino-benzoic acid ester (Tricaine) was used to anesthetize animals for imaging. After anesthetization, they were covered in 0.8% low-melting point agarose in glass-bottomed 35 mm Petri dishes and mounted on their right side. Images were captured using a spinning disk confocal microscope custom build by 3i technology© with: Zeiss Axio Observer Z1 Advanced Mariana Microscope, X-cite 120LED White Light LED System, filter cubes for GFP and mRFP, a motorized X,Y stage, piezo Z stage, 20X Air (0.50 NA), 63X (1.15NA), 40X (1.1NA) objectives, CSU-W1 T2 Spinning Disk Confocal Head (50 uM) with 1X camera adaptor, and an iXon3 1Kx1K EMCCD camera, dichroic mirrors for 446, 515, 561, 405, 488, 561, 640 excitation, laser stack with 405 nm, 445 nm, 488 nm, 561 nm and 637 nm with laser stack FiberSwitcher, photomanipulation from vector© high speed point scanner ablations at diffraction limited capacity, Ablate!TMª Photoablation System (532 nm pulsed laser, pulse energy 60J @ 200 HZ). Time-lapse images were collected every 5 min for 24 hr. Images were processed using Slidebook software and ImageJ. Only brightness and contrast were adjusted for all images.

#### Laser-Induced Spinal Avulsion

Animals were first mounted in 0.8% agarose as described above. One avulsion was made per animal except in Figure 4. Injuries were consistently made in the trunk of the animal at DRG 10–14. The specific site of laser injury was chosen by following dorsal projections from the DRG into the spinal cord through the z-plane. An area of clear axon fluorescence in the PNS was marked and brought into focus. The image window was then adjusted 1um laterally out of the z-plane to avoid spinal injury, and a double-click on the axon using a 4 μm selection tool created the injury. All laser parameters used are relative to our confocal microscope: laser power (1), raster block size (1), double-click rectangle size (8), double-click repetitions (4).

#### Chemical Treatments

The chemical treatments used in this study are taxol (Acros), GM6001 (Santa Cruz Biotechnology), and SU6656 (Santa Cruz Biotechnology). Stock solutions of these reagents were stored at −20°C. The taxol stock solution was stored at 2.2 mM, the GM6001 stock solution was stored at 1.0 mM, and the SU6656 stock was stored at 375mM - all in DMSO. Treated animals were exposed to the chemical directly following injury (expect in Figure 2F where animals were exposed at varying time-points after injury) at concentrations of 22 mM for taxol, 1 μM for GM6001, and 3μM diluted in egg water (Nichols and Smith, 2019). DMSO-treated animals were exposed to 1% DMSO in egg water.

#### Immunohistochemistry

The primary antibodies used in this study are Zrf-1 (1:100, mouse, ZIRC) and phospho-p44/42 MAPK (Erk1/2) (Thr202/Tyr204) (1:500, rabbit, Cell Signaling). Alexa Fluor 647 goat anti-mouse (1:600, Thermo Fisher) and Alexa Fluor 647 goat anti-rabbit (1:600, Thermo Fisher) were used as secondary antibodies. Fish were stained in 4% PFA in PBST (PBS, 0.1% Triton X-100) for 3 hr at 25°C. The fixed fish were then washed with PBSTx (PBS, 1% Triton X-100) for 5 min followed by washes in DWTx (PBS, 1% DMSO, 1% Triton X-100) and acetone for 5 min each at 25°C. The samples were then washed in acetone for 10 min at −20°C. Fish were exposed to three washes of PBSTx for 5 min each at 25°C followed by 1 hr of 5% goat serum in PBSTx at 25°C. The fish were then incubated with the primary antibody in PBSTx and 5% goat serum for 1 hr at 25°C followed by 4°C overnight. The following day, fish were washed three times with PBSTx for 30 min and once in PBSTx for 1 hr, all at 25°C. The fish were then incubated with the secondary antibody in PBSTx and 5% goat serum at 25°C for 1 hr followed by 4°C overnight. The next day, the samples were washed three times in PBSTx for 30 min at 25°C. Stained fish were then stored in PBS and 50% glycerol at 4°C until imaging. Stained fish were mounted and imaged using the same techniques described above.

#### Molecular Biology

Creation of psELN05 *(uas:ca-src-mcherry + clmc2:gfp*), *psELN06 (uas:src-mcherry + clmc2:gfp), and psELN10 (gfap:ca-srcmcherry)* were generated using the LR Clonease II Plus (ThermoFisher) using zebrafish compatible Tol2 constructs. To express the UAS promoter, *p5e-UAS and p5e-gfap* vectors were used (Don et al., 2017). *pMe-Src and pMe-CASrc* vectors were used to express the two Src forms (Seiler et al., 2012) and were fused to mCherry using the *p3e-mCherrypA* construct. Multisite gateway recombination of these constructs was accomplished using *pDestTol2CG2* (Kwan et al., 2007). The *pMe-Src* and *pMe-CASrc* constructs were generous gifts of Christoph Seiler. A *uas:syn-gfp* plasmid was also used to generate animals for the synapse analysis (Hines et al., 2015).

#### Expression of Plasmid Constructs

To express the uas:src-mcherry, *uas:ca-src-mcherry, gfap:ca-src-mcherry, uas:syn-gfp* constructs, the DNA was diluted in water to a 75 ng/ml working solution. For injections, the working solution was diluted further to 12.5 ng/μl in water with 25 ng/ml of *pCS2FA-transposase* RNA in water. This solution was injected into single-cell embryos. For *uas:src-mcherry* and *uas:ca-src-mcherry* injections, *Tg(sox10:gal4); Tg(uas:lifeac-gfp)* animals were used. For *gfap:ca-src-mcherry* injections, *Tg(ngn1:gfp)* animals were used. For *uas: syn-gfp* injections, *Tg(sox10:gal4)* animals were used. At 3dpf, injected animals were assayed for transgenic background fluorescent expression. One DRG per animal was imaged to confirm mCherry expression and then subjected to avulsion-like injury. Time-lapse imaging was then taken of avulsed DRG. Following imaging, fish were immediately fixed and stained for GFAP to visualize the glia limitans.

#### Behavioral Assay

One day after injury, individual zebrafish were removed from their 23°C husbandry temperature, immersed in 4°C or 23°C and imaged with a Zeiss Axiozoom microscope and a monochrome camera controlled by Zen software. Movies of the 20 s immediately following exposure to 4°C or 23°C water were taken at 20 frames per second. These videos were compiled and analyzed for shivering behaviors. Shivering was defined as bilateral movement of the larval tail that did not result in the animal’s forward locomotion. All analysis was completed by a researcher blind to the experimental paradigm the animal was exposed to. Quantified numbers and percent of time shivering was classified into four categories of behavioral responses: typical (3+ shivers and greater than 12% shivering), stunted (7%–12% but 1–3 shivers), moderate (1–3 shivers and 1%–7%), or absent (no shivering).

### QUANTIFICATION AND STATISTICAL ANALYSIS

Slidebook software was used to generate composite z-images and y-orthogonal images. The mean and SEM are shown for all representations of error. Values for n reflect the number of DRG quantified unless otherwise noted. Intensity quantifications were completed using ImageJ and cell tracing through time-lapse movies was performed using the MTrackJ plugin in ImageJ. GraphPad Prism software was used to generate all graphs in this study and for all statistical analyses. Additional methods to determine if the data met assumptions of the statistical test were not used. Statistical tests, sample size and definition of precision measurements for each experiment can be found in the main text and figure legends for all experiments.

### DATA AND CODE AVAILABILITY

All data generated in this study are included in the article. No code was used or generated in this study.

## Supplementary Material

1

2

3

4

5

6

## Figures and Tables

**KEY RESOURCES TABLE:** 

REAGENT or RESOURCE	SOURCE	IDENTIFIER
Antibodies		
Zrf-1 (anti-GFAP, mouse monoclonal)	ZIRC	RRID: AB_10013806
Phospho-p44/42 MAPK (anti-ERK1/2 Thr202/204, rabbit monoclonal)	Cell Signaling	Cat #: 9101S; RRID: AB_331772
Alexa Fluor 647 goat anti-mouse	Thermo Fisher	Cat #: A-21235; RRID: AB_2535806
Alexa Fluor 647 goat anti-rabbit	Thermo Fisher	Cat #: A-20991; RRID: AB_2535814
Bacterial and Virus Strains		
Mix & Go Competent Cells	Zymo Research	Cat #: T3007
Chemicals, Peptides, and Recombinant Proteins		
Paclitaxel	Acros	Cat #: 328420050; CAS: 33069–62–4
GM6001	Santa Cruz Biotechnology	Cat #: sc-203979; CAS: 142880–36–2
SU6656	Santa Cruz Biotechnology	Cat #: sc-203286; CAS: 330161–87–0
LR Clonase II Plus	Thermo Fisher	Cat#: 12538120
Experimental Models: Organisms/Strains		
AB	ZIRC	Wildtype zebrafish strain
Zebrafish: *Tg(ngn1:gfp)*	McGraw et al., 2008	ZFIN: ZDB-ALT-090806–1
Zebrafish: *Tg(sox10:gal4)*	Hines et al., 2015	ZFIN: ZDB-FISH-150901–5454
Zebrafish: *Tg(uas:lifeact-gfp)*	Helker et al., 2013	ZFIN: ZDB-FISH-150901–1674
Zebrafish: *Tg(gfap:nsfb-mcherry)*	Johnson et al., 2016	ZFIN: ZDB-ALT-160630–2
Zebrafish: *Tg(uas:syn-gfp)*	Heap et al., 2013	ZFIN: ZBD-ALT-130702–2
Recombinant DNA		
*p5E-UAS*	Kwan et al., 2007	http://tol2kit.genetics.utah.edu/index.php/Main_Page
*p5e-gfap*	Don et al., 2017	
*pME-Src*	Seiler et al., 2012	
*pME-CA-Src*	Seiler et al., 2012	
*p3E-mCherry-pA*	Kwan et al., 2007	http://tol2kit.genetics.utah.edu/index.php/Main_Page
*pDestTol2CG2*	Kwan et al., 2007	http://tol2kit.genetics.utah.edu/index.php/Main_Page
*psELN05 (pTol2-uas:CA-Src-mCherry, cmcl2:gfp)*	This paper	N/A
*psELN06 (pTol2-uas:Src-mCherry, cmcl2:gfp)*	This paper	N/A
*psELN10 (pTol2-gfap:CA-Src-mCherry, cmcl2:gfp)*	This paper	N/A
Software and Algorithms		
ImageJ	NIH	RRID: SCR_003070; https://imagej.nih.gov/ij/
MTrackJ	Image Science	https://imagescience.org/meijering/software/mtrackj/
Slidebook	3i	RRID: SCR_014300; https://www.intelligent-imaging.com/slidebook
Prism	GraphPad	RRID: SCR_002798; https://www.graphpad.com/

## Highlights

- Visualization of regenerating nerves in zebrafish model of avulsion-like injuries
- Regenerating DRG sensory axons do not invade back into the spinal cord
- Stabilization of invasion components induces regeneration
- Regenerated axons restore neural circuits and behavior by 1–2 days after injury
