# Supplementary material for: Functional Regeneration of the Sensory Root via Axonal Invasion
Source: Cell Rep. Author manuscript; Available in PMC 2020 Feb 3. (PMC6996490; doi:10.1016/j.celrep.2019.12.008)
Supplement: 1 [file NIHMS1548563-supplement-1.pdf]

Cell Reports, Volume *30*

Supplemental Information

Functional Regeneration of the Sensory Root via Axonal Invasion

Evan L. Nichols and Cody J. Smith

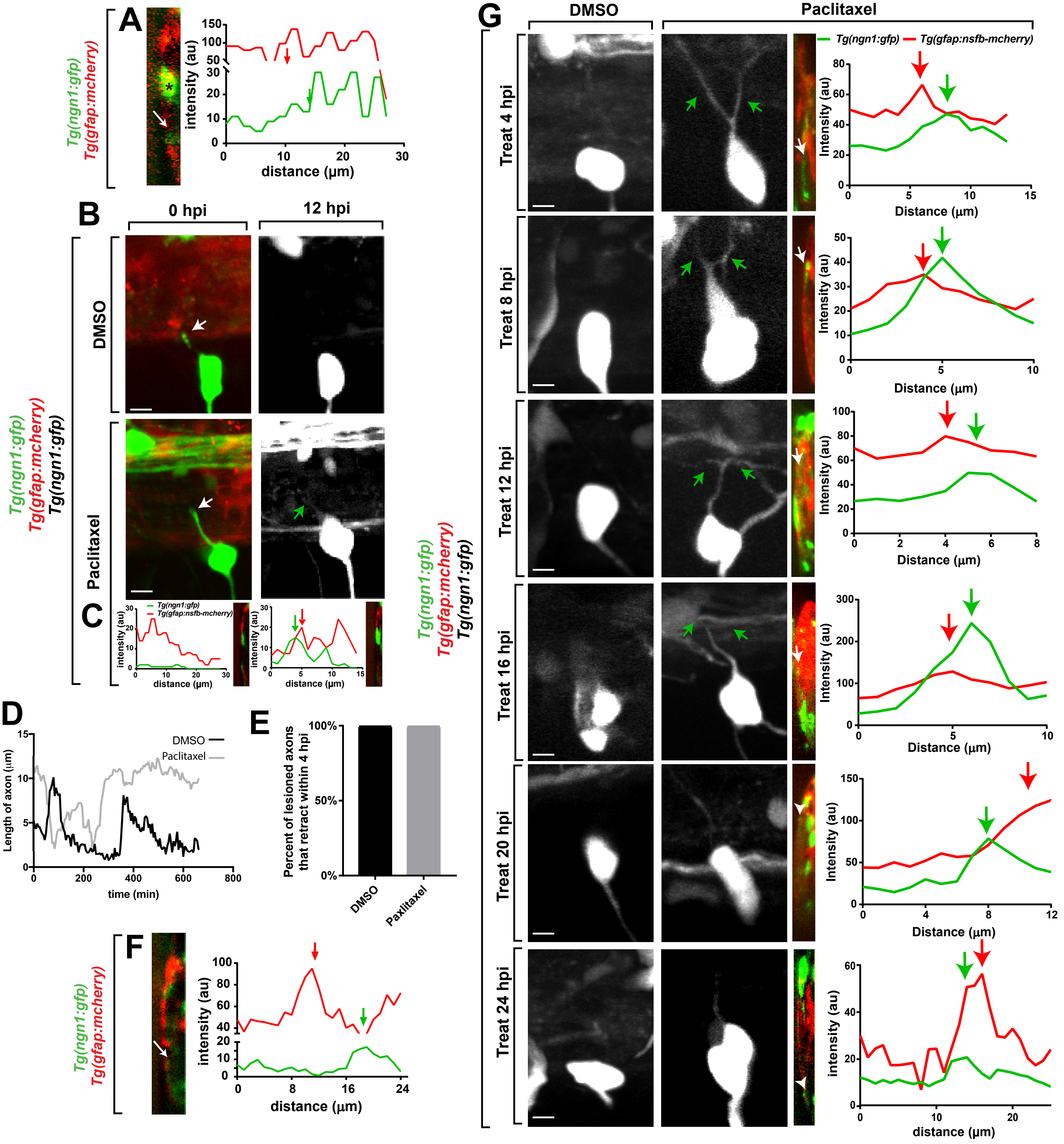

Figure S1. Taxol promotes regeneration as the axon re-extends, related to Figure 1. (A,F). Y-orthogonal images of the animals from Figure 1I treated with taxol at 3dpf (A) and 5dpf (F). White arrows denote GFP puncta within glia limitans. Graphs show representative tracings of GFP (regenerated axon) and mCherry (glia limitans) from the y-orthogonal images. Green arrows denote regenerated axon and red arrows denote glia limitans. Asterisk denotes neuron in the spinal cord. (B). Z-projection time-lapse images of *Tg(ngn1:gfp); Tg(gfap:nsfb-mcherry)* animals at 0 and 12hpi treated with DMSO or taxol. White and green arrows denote regenerating growth cone. (C). Y-orthogonal images of animals from (B) treated with taxol. Graphs show representative tracings of GFP (axon) and mCherry (glia limitans) from y-orthogonal images. Green arrows denote axon and red arrows denote the glia limitans. (D). Length of re-extending axons treated with DMSO and taxol after injury. (E). Percent of axons treated with DMSO and taxol that retract to the DRG after injury. (n=6 DRG per treatment) (G). Left: Z-projection images of *Tg(ngn1:gfp); Tg(gfap:nsfb-mcherry)* animals at 48hpi and treated with DMSO or taxol at 4, 8, 12, 16, 20, and 24hpi. Green arrows denote the regenerated axon. Right: Y-orthogonal images of animals treated with taxol. Graphs show representative tracings of GFP (axon) and mCherry (glia limitans) from the y-orthogonal images. Green and white arrows denote axon and red arrows denote the glia limitans. Scale bar is 1 µm.

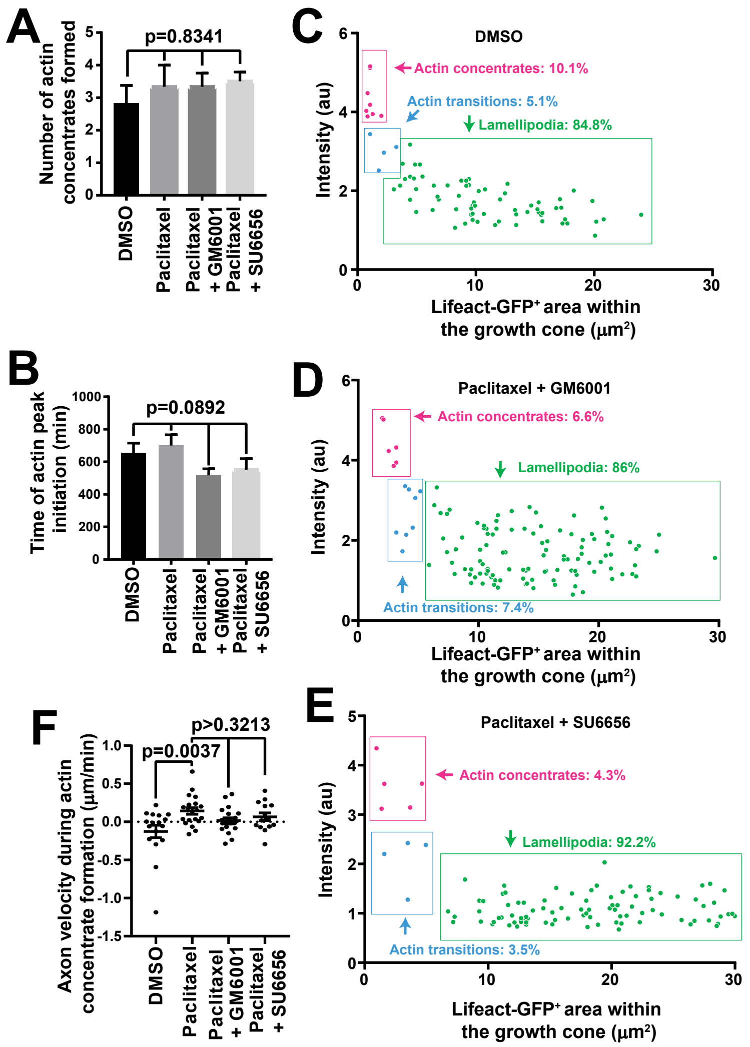

Figure S2. Taxol treatment favors actin organization into concentrates, related to Figure 2. (A-B). Graphs of the number of actin concentrates (A) and time of actin concentrate initiation (B) DMSO (n=7), taxol (n=6), taxol+GM6001 (n=6), and taxol+SU6656 (n=4) treated animals. (C-E). Representative scatterplots comparing growth cone area and average Lifeact-GFP intensity at each time point during regeneration in an axon treated with DMSO (C), taxol+GM6001 (D), and taxol+SU6656 (E). Each time point is represented by a point. Pink points denote actin concentrates. Blue points denote actin transitions. Green points denote dispersed actin. (F). Graph of average growth cone velocity during actin concentrate formation. DMSO-treated axons display negative velocities in the presence of actin concentrates, while taxol and cotreated axons do not. (A,B,F) use Tukey's HSD.

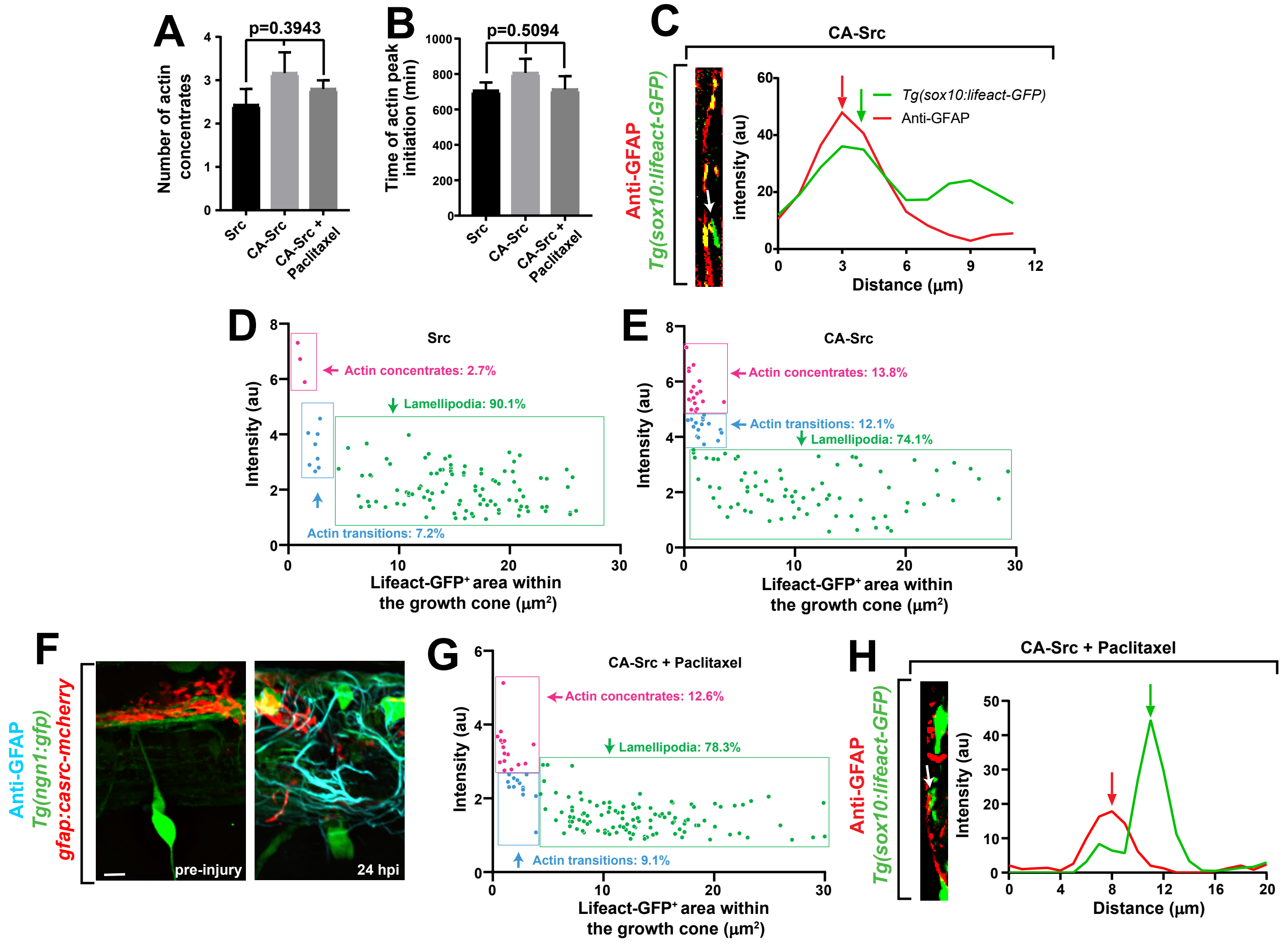

Figure S3. CA-Src expression in DRG cells favors actin organization into concentrates, related to Figure 3. (A-B). Graphs of number of actin concentrates (A), and time of actin concentrate initiation (B) in DRG expressing Src, CA-Src, and CA-Src+taxol. CA-Src and CA-Src+taxol axons demonstrate an increased duration of actin concentrates. (n=6 Src and CA-Src, n=5 CA-Src+taxol) (C,H). Y-orthogonal image of the animals in Figure 3B (C) and Figure 3C (H) stained for GFAP. White arrow denotes GFP+ puncta within the glia limitans. Graphs show representative tracings of regenerated axon (green) and glia limitans (red) from the y-orthogonal image. The regenerated axon is inside the spinal boundary. Green arrow denotes regenerated axon and red arrow denote the glia limitans. (D,E,G). Representative scatterplots comparing growth cone area and average Lifeact-GFP intensity at each time point during regeneration in an axon expressing Src (D), CA-SRC (E), and CA-Src+taxol (G). Each time point is represented by a point. Pink points denote actin concentrates. Blue points denote actin transitions. Green points denote dispersed actin. (F). Z-projection images of a *Tg(ngn1:gfp); gfap:-CA-src-mcherry* animal before injury and 24hpi. At 24hpi, the animal was fixed and stained for GFAP. At 24hpi, the neuron has retracted its axon consistent with failed regeneration. (A,B) use Tukey's HSD. Scale bar is 10 µm.

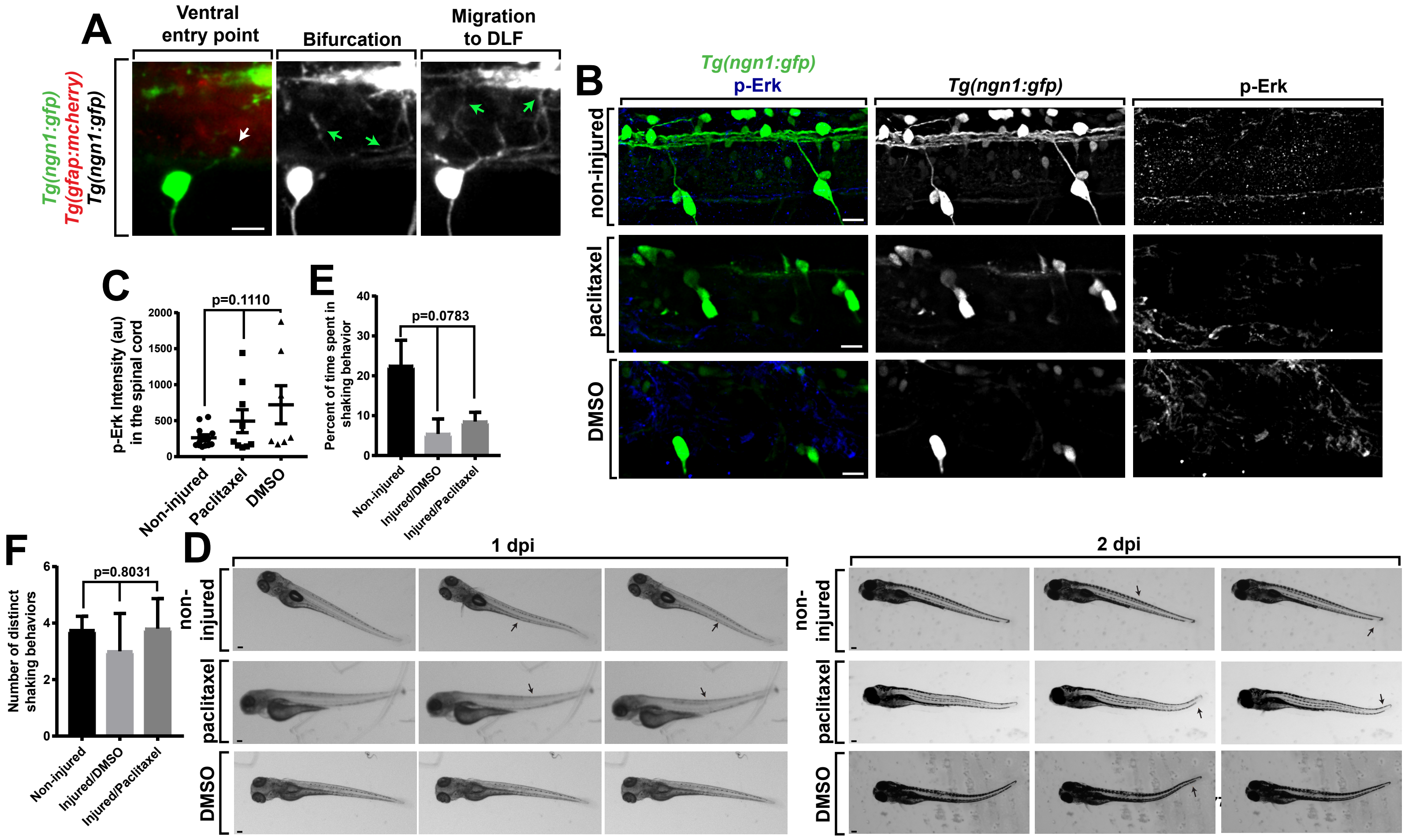

Figure S4. Dynamics of circuit and behavioral functioning following multiple avulsions, related to Figure 4. (A). Z-projection time-lapse images of a *Tg(ngn1:gfp); Tg(gfap:nsfb-mcherry)* animal treated with taxol following avulsion-like injury showing atypical DRG spinal entry and navigation of the axon to the DLF. White and green arrows denote regenerating growth cone (B). Z-projection images of T*g(ngn1:gfp)* animals stained for p-Erk after exposure to 25°C and without avulsions, 8 consecutive avulsions and DMSO treatment, or 8 consecutive avulsions and taxol treatment. (C). Average intensity of pErk in the spinal cord of after exposure to 25°C. (n=5 animals per treatment) (D). Time-lapse mages of animals without avulsions, 8 consecutive avulsions and DMSO treatment, or 8 consecutive avulsions and taxol treatment at 1 dpi and 2 dpi. Black arrows denote shivering behavior. (E-F). Percent of time in shaking behavior (E) and number of shaking behaviors (F) at 2 dpi. (n=5 animals per treatment) (A,C,E-F) use a one-way ANOVA. Scale bar is 1 µm in (B) and 0.1 mm in (D).
